# Supplementary material for: Multimodal neuroimaging markers of variation in cognitive ability in older HIV+ men
Source: PLoS One. 2021 Jul 27;16(7):e0243670. doi: 10.1371/journal.pone.0243670 (PMC8315526; doi:10.1371/journal.pone.0243670)
Supplement: Attachment — Submitted filename: Response to Reviewers -Multimodal neuroimaging.. March2021.docx [file pone.0243670.s001.docx]

Thank you for considering our manuscript for publication and for providing these detailed and helpful reviews. We have extensively revised the manuscript to address the points raised by the reviewers. We have addressed the comments of the reviewers below, point by point, and in the manuscript. All changes are in blue in the manuscript.

**Review Comments to the Author**

**Reviewer #1:** This exploratory study examines 66 men with HIV on cART utilizing EEG and sMRI. The authors use two cognitive tasks during EEG acquisition: a simon task, and an auditory oddball task. They go on to examine time-domain signal properties and confirmatory perform source localization on their EEG data, and extract subcortical volumes from their sMRI data. They find that P300 is related to global cognition, and P300 from the oddball is related to CD4 nadir. Strengths include multimodality (although this only uses a small subsample of n=27) and examination of two EEG tasks. However, their clear limitation is a lack of an uninfected control group. This limits their HIV related conclusions to the relationship between P300 and CD4 nadir. Further issues include only examining men, limited research question examining small variation in cognitive function, and an analysis that appears somewhat selective.
The goal of this study was to examine the neural basis of individual differences in cognitive ability in people with HIV, both to shed further light on the pathophysiology and to suggest high-yield candidate biomarkers for cognition in the normal-to-mildly impaired range of cognitive performance that is the greatest clinical diagnostic challenge in well-treated HIV in our setting. Addressing these aims does not require an HIV- control group. Indeed, we are interested in non-HIV-specific explanations for cognitive variability in people living with HIV, as many of these might be remediable. Designs comparing HIV+ and HIV- groups have their own pitfalls, given the challenges of deciding which variables should be ‘controlled for’ in the control group. We have clarified these goals in the revised paper and added an explicit discussion of this point in the Limitations section of the Discussion.

**Abstract**
The abstract sample size is misleading, especially for the multimodal portion, which they state 54 completed MRI, but ultimately only used 27 of those participants.

The number reported in the abstract corresponds to the number of men who completed the EEG study. We have now clarified the number with usable EEG data (N=66) in the Abstract. We reported the sample following the [CONSORT](http://www.consort-statement.org/) (Consolidated Standards of Reporting Trials) guidelines and included a CONSORT flow diagram (Figure 1) to display the progress of all participants through the study. Of the 66 participants with EEG data, 43 also had structural MRI. We used the MRI data from all 43 for source localization. We focused on the 27 people who had usable EEG data from both tasks and structural MRI only for the exploratory analysis of the relationship between regional brain volumes and P300 waveforms. This was not the main goal of the paper, but it seemed to us important to carry out at least an exploratory analysis, given these rich multimodal data. We have revised the Figure legends, Methods and Results to make the samples available for each analysis clearer.

“This is the first study to combine structural and functional imaging in an overlapping sample to address the neural circuits related to cognitive dysfunction in HIV.” Although relatively few studies exist, this isn’t the first. The following is just a selection of studies all have structure, function and cognitive testing in a single sample: (Cole, James H., et al. Clinical Infectious Diseases 66.12 (2018): 1899-1909; Samboju, Vishal, et al. NeuroImage: Clinical 20 (2018): 327-335; Wilson et al., Human brain mapping 36.3 (2015): 897-910.)

We believe we are the first to combine EEG as a functional brain measure with structural MRI and have revised this section to clarify what we meant by functional imaging. Our findings suggest that EEG, in addition to previously reported other functional imaging techniques may be a useful approach for characterizing subtle brain dysfunction underlying milder cognitive impairment in HIV. To our knowledge no other studies have evaluated neural circuits using EEG and structural MRI in a sample of cART treated HIV + individuals with well controlled infection and mild cognitive impairment. EEG is cheap and widely available indicators of neural function that could prove relevant to monitor for cognitive decline as well as response to treatment or rehabilitation, especially in resource-limited settings where HIV is endemic and equipment and expertise may not be available for functional MRI or MEG. We have clarified this in the revised manuscript.

**Introduction**

While cognitive impairment is certainly still an issue in older men with HIV, I think the authors should acknowledge some of the recent literature calling into question whether the prevalence of HAND has been overestimated (Su et al., Aids 29.5 (2015): 547-557; Zheng et al., Aids 33.14 (2019): 2115-2124; Meyer et al., Neuroepidemiology 41.3-4 (2013): 208-216).

We agree that the conventional prevalence estimates are likely too high, due to several factors, not least: selection bias. We have clarified this in the Introduction.

I’m not sure I would categorize an auditory oddball task as a “attention and working memory-requiring categorization task.” Although true, that is likely true of any task, and the inherent contrast of oddball tasks doesn’t probe working memory, moreso vigilance.

We do not claim that the oddball task is uniquely requiring working memory and attention, but as the reviewer points out, it likely requires those abilities. The oddball task requires one to hold in mind the preceding auditory tone and compare it to the current tone. The Simon task, for example, does not require comparing the preceding stimulus to the current stimulus thus, conventionally, is not considered a test of working memory. We now cite more fully the literature supporting the claim that oddball tasks tap working memory and attention. We agree that vigilance is also needed, although it seems to us more generic (e.g. likely to be required in any evoked potential study which necessarily requires many repetitive trials). In any case, these characterizations are not critical to the interpretation of this study. It is enough to propose that the two tasks likely involve partially distinct neural circuits, a claim borne out by an extensive literature (now quoted more fully) and our own findings.

The authors state their goal was to “provide evidence of the neural mechanisms underlying variation in the mild cognitive impairment to normal range.” Further justification for this aim is needed as I’m left wondering why mild (asymptomatic) to normal fluctuations in cognitive function is relevant, as opposed to more severe cognitive impairment. Their lone justification is in their first paragraph stating “impairments can nonetheless limit everyday function.” I encourage them to expand upon this further, potentially adding more about the papers they cite relating cognitive function to medication adherence and quality of life.

In the clinic, cognitive impairment in the mild-to-normal range is the most frequent neurocognitive issue in those with viral suppression. Dementia is generally easy to recognize and is also mercifully uncommon. The milder cognitive impairment range poses the most substantial diagnostic and treatment challenges. We and others have shown the real-world impact (on work status, adherence, and quality of life) of cognitive difficulties in this range. We have added more details.

**Methods**
It seems inappropriate to have excluded women from the study, especially when data were available for 6 women. While the authors state a sample of 6 would be insufficient to examine sex effects, the relevant research question here does not necessitate the exclusion of participants based solely on sex.

The decision to exclude the data from the 6 women was based on the following: First, previous analyses from the Brain Health Now cohort, n= 856 (the parent study from which our sample here is drawn) showed that results from women were systematically different from those of men in many regards (Relationships between cognition, function, and quality of life among HIV+ Canadian men, NE Mayo, MJ Brouillette, SC Scott, M Harris, F Smaill, G Smith, R Thomas, ...Quality of Life Research 29 (1), 37-55). This is not surprising in the Canadian context, where there are many substantial demographic and social differences between men (mainly white, well-educated men who have sex with men) and women (mainly recent immigrants from the Caribbean or sub-Saharan Africa with language and cultural barriers, and limited education) with HIV. Given this strong prior, we do not think it is wise to include such a small sample of women in the analysis. In our view, this will introduce noise while adding only the illusion of generalizability.

We completely agree that it is important to make this limitation very clear. We have emphasized it in the Discussion and taken pains to remind the reader throughout that the findings here cannot be generalized to women.

In on-going work, we are recruiting a large sample of women to better understand differences and commonalities at clinical-demographic and brain levels.
The entire analysis is based on a global composite of the B-CAM. They state 53 participants had standard neuropsychological testing. What to their results look like examining these participants and their standard neuropsychological testing data?
We have shown that the B-CAM continuous score is a mathematically meaningful measure of overall cognitive performance in people with HIV (Koski,et al, 2011). We have also validated the B-CAM against the many existing methods of operationalizing the Frascati classification of HAND (Brouillette, in press). The B-CAM is as useful as any of these methods for predicting important real-world outcomes, such as work status and work productivity. There is very little classification agreement amongst the existing neuropsychological classification approaches (and thus, unsurprisingly, between the BCAM and these various classification approaches), based on our work in the full Brain Health Now sample (N=856). This makes validation against neuropsychological testing a highly problematic undertaking. Given our own work demonstrating the methodological difficulties with current HAND classification methods, we don’t think that adding yet another subset analysis to this manuscript is likely to shed additional light, and we fear it will add further complexity to an already dense paper. We have indicated the mean BCAM score for those with or without HAND in those who had neuropsychological testing in the present sample, as a guide to the reader more familiar with the conventional neuropsychological approach.

The authors cite away the entire structural analysis. A brief summary of the segmentation in this manuscript would be helpful.

We have added a description of the analysis.

Was a variable inter-stimulus interval used? This is important considering the prestimulus baseline.

Thank you for pointing out the need for this information. Yes, the time between the offset of one stimulus and the onset of the next varied. We added this to the Methods. Note that in the case of the oddball task, the task design was a non-traditional one, where the expectation of the stimulus onset was set by the probability of the 3rd tone of a series of 4 being a standard or deviant tone. We included a note on this in the Discussion as well.
Results
The sample had an average B-CAM of 20.7. Looking into the B-CAM, it appears the maximum score is a 24. There may be ceiling effects at play. Can the authors provide a plot showing the distribution of B-CAM scores?

The B-CAM score ranges from 0 to 41, but in this sample the observed range was 12-31. We have added this information to the manuscript. There were no ceiling or floor effects, and as mentioned in the manuscript, the scoring of the B-CAM is mathematically meaningful, making it particularly well-suited to linear regression. Indeed, we developed the B-CAM to address ceiling effects in other more widely used brief cognitive assessment tools in similar clinical samples (see e.g. Koski et al, 2011; Brouillette et al. AIDS, 2015).

It is stated that evoked potentials displayed in Figures 2 and 3 represent grand averages, which would imply an average across the sample. It would therefore be helpful to show error bars on these timeseries.

Thanks for this comment. Variance around the means of all key ERP measures is shown by standard deviations provided in Tables, 1, 2 and 3.

Much of the data is thrown out early on in the analysis. Only large deviant trials and incongruent trials are used for the HIV analysis based on the relationship between behavior on these trials and BCAM scores. I believe the more intuitive approach would be to utilize contrasts between conditions. The authors even show behavioral effects between conditions in their repeated measures ANOVAs. Why then are these condition effects suddenly ignored when examining neural data?

We took a step-wise approach to the analysis of this rich data set, guided by the study aims and a previous systematic review which yielded specific hypotheses about the EEG effects. We reported the contrasts between conditions in the neural data: see section ***ERPs in response to Simon and oddball tasks*** where we describe the effects of trial type, i.e., conditions on the amplitude of the ERPs. We show these effects for both tasks, validating the condition effects as expected from the literature.

The aim of this study was to provide evidence for the neural mechanisms underlying variation in cognitive ability amongst participants. Thus, we chose multiple linear regression as the most pertinent analytic approach.
There appears to be a typo, with effect of P300 amplitude stated twice with differing p values: “The amplitude of the P300 evoked by the oddball task at the cluster of electrodes around Pz was significantly related to B-CAM (F (3, 50) = 4.73, p = 0.005), with a marginal effect of P300 amplitude (p = 0.1) and age (p = 0.04).”
As we indicated at the beginning of the paragraph the reviewer mentions, the results reported are from multiple linear regression models. As such, we report first the results of the linear model (statistical significance of the prediction of the B-CAM score based on the two predictors of interest), and subsequently the contribution of each of the two variables of interest (P300 amplitude and age) in explaining variation on the B-CAM score. These results are reported following APA format. If the Editor prefers, we could modify the format.

Given the use of a global B-CAM score, can the authors provide any insight into whether certain cognitive domains drove these differences?

In other work, we have shown that cognition can be assessed as a single construct in HIV; we have shown empirically that apparent domain effects are largely driven by task difficulty differences, rather than by selective impairments in domains as might be seen in a more focal brain disorder. The B-CAM was developed using item-response theory as a measure of this latent construct. We clarify this in the manuscript and provide additional references.

The source localization analysis seems more supplementary. It does not give added information besides that the P300 for the two tasks are not perfectly identical when source localized, which I wouldn’t expect in the first place given the large differences in the tasks. The maps actually looked more similar than I would have expected. Why examine only the 27 participants that completed both tasks and MRI, when separate correlations are run for each task?

We agree that the source localization analysis is supplementary and is provided to highlight the difference between earlier auditory and visual activity and the later auditory and visual cognitive processing. The source localization used all available data. However, when analysing the relationship between subcortical volumes and P300 amplitudes in both tasks, we were careful to include only participants that had completed both tasks and had available MRI data. We conducted this exploratory analysis only on participants with complete data to avoid spurious effects driven by particular participants not present in both sub-samples.

Given the relevant results are derived from regressions and correlations, scatterplots with best fit lines for all significant results are needed to get a better sense of the distributions of the variables.

When presenting the results of the multiple linear regressions we included the standard errors, parameter estimates, i.e, beta coefficients as well upper and lower bounds of the 95% confidence interval in the tTables to provide a sense of the variable distributions and effect sizes to the readers. We believe that for the number of variables we have in this study a table is more efficient than multiple graphs to understand the effect sizes and distributions. Following the APA guidelines for reporting results, we do not find it appropriate to re-report this information in Figures. We present below, added variable (partial regression) plots depicting the relationships of P300 amplitude with cognitive ability and infection severity (nadir CD4 count) for the reviewer to visualize the distribution and relationship between variables of interest

**Auditory Oddball p300 -added variable plots**

**Simon Task p300 -added variable plots**

Discussion
The authors state: “provided evidence that early sensory and cognitive processing stages in both tasks did not relate to overall cognitive ability…” Technically their statistics did not test for support of the null hypothesis, but just failed to reject the null hypothesis. Baysean statistics could be used to show that these variables in fact do not relate to cognitive ability ect.

We agree and have rephrased this sentence in the Discussion.

Additionally they state: “Here, in a larger sample, using cortical source estimation we show that these earlier processing stages in auditory and visual modalities are relatively spared.” This is not accurate because they do not have an uninfected control group to compare to. They also do not use source estimation other than to qualitatively compare P300 of the two tasks.

Thank you for highlighting this, indeed the conclusion we draw on earlier processing stages is from the multiple linear regressions of the amplitude of the N100 and N200, not from the source estimation, and the word ‘spared’ is not what intended to convey. We have rephrased these two sentences in the Discussion.
Further acknowledgment of other health comorbidities needs to be addressed in the limitations, as even factors such as adipose tissue can be related to neural alterations that might have otherwise been attributed to HIV (Lake et al., 2017. J Neurovirol;23(3):385-393). I use this as an example, as a multitude of other comorbidities are not explicitly controlled for in this study, and may therefore be contributing factors.

We now further discussed the potential effects of comorbidities and complications of chronic HIV.

The factors we chose to study were based on evidence from previous studies in the full Brain Health Now cohort. The number of participants in the current study limits the statistical power of our analysis in the case of multiple comparisons. Thus, we limited the non-hypothesis-driven analysis. We address this now directly in the manuscript.

Regarding the effects of adipose tissue, abdominal obesity is included as a risk factor for metabolic syndrome in this study, which was included in our analysis and did not show any effects. We added this information in the manuscript.

***********************

**Reviewer #2:** This is an interesting yet straightforward investigation of the association of event related potentials, recorded during an auditory oddball task and visual Simon task, with nadir CD4 count and cognitive ability among older HIV-1 seropositive men. There is, unfortunately, no HIV-1 seronegative control group and no women were included. But, the findings are consistent with the findings of similar studies published previously. I think that this MS is clearly written. It includes appropriate analyses. The conclusions are reasonable and justified by the findings.

Thank you for this positive assessment.

I do have a few minor comments and suggestions:
1. Both the auditory oddball and Simon tasks used presently are different in format from what we usually see. Here, the auditory task is not a running series. It presents the deviant stimulus at a predictable time (#3 in a series of 4) and therefore produces a smaller P300 than we typically see in this literature. This should be pointed out to readers not familiar with ERP methods. Also, the Simon task is not presented in a typical manner.

Thank you for pointing this out, we have added a note on the multiple oddball task in the Discussion. Regarding the Simon task we used for this study. There are more variations on this task than the oddball task, but the one we selected has been widely used. We reference the studies that we relied on in programming the Simon task used here.
2. It would be useful to know more about the participants. What was the race/ethnic composition of the sample? Instead of telling us the percentage who attended college, please report the number of years of education and the variability? Do you have any information about family history, substance abuse history, or history of childhood ADHD or conduct problems? As the authors know, all of these factors have been shown to affect P300 and complicate the interpretation of HIV effects on P300.

We did not characterize race or ethnicity, or family history or personal history of psychopathology such as ADHD in this sample. People with current substance dependence were excluded. As we indicate in the paper, over half the sample attended university. There were very few people who had less than a high school education, too few to draw conclusions about finer grained educational effects, especially given the overall sample size. We have clarified this in the manuscript.

The reviewer brings up many potentially interesting variables, and we are familiar with the literature he or she references, but these variables are beyond the scope of our current study. We are studying some of these in relation to cognition in the larger Brain Health Now cohort, where adequate power to definitively test for effects on cognition is more likely. For example, in this non-substance dependent cohort, we have not observed substantial effects of recreational drug use on cognition.

3. It is not surprising that anxiety and depression were not related to ERPs in this sample. First, it is a prospectively followed cohort that may be healthier and therefore more cooperative than we see in cross-sectional studies. Second, no women were studied--rates of anxiety and depression are generally lower in men.

The cohort from which these participants were drawn was systematically sampled from HIV speciality clinics in Montreal. We have assessed selection bias in the main cohort (and published on it). In fact, people with fewer cognitive or mental health concerns were less likely to join the cohort than those with brain health concerns. Clinically significant anxiety and depression are common in the cohort as a whole and there is a range of such symptoms in the sample studied here, as summarized in Table 1. For example, the mean HADS anxiety score in this sample was 7, at the cut-off for clinically significant anxiety.

4. In addition to citing your recently published review of P300 (and other ERP components) in HIV, you might cite a few of the original primary sources.

When a systematic review exists, current recommendations are not to cite a selection of the original sources, as this will unavoidably be a biased selection. This is why we undertook and published a systematic review.
